# AD pathology in LBD – Implications for Clinical Trials

**DOI:** 10.1002/alz.091281

**Published:** 2025-01-09

**Authors:** Bhavana Patel

**Affiliations:** ^1^ University of Florida, Gainesville, FL USA

## Abstract

Alzheimer’s disease (AD) is a common copathology in Lewy body dementia (LBD). The presence of AD is associated with a distinct clinical presentation, faster progression, and shorter survival in LBD. However, the relationship between‐alpha synuclein and AD remains incompletely understood. The objective of this session is to discuss the challenges that this poses to clinical translation of approved therapies for AD and LBD‐specific clinical trial design.

First, we will discuss the impact of the significant advances in the treatment of AD that have occurred in the past years. Specifically, the publication of evidence supporting the use of anti‐amyloid monoclonal antibodies as disease‐modifying therapies for AD leading to the approval of three different therapies by the Food and Drug Administration (FDA). Currently, Lewy body dementias are excluded from the appropriate use recommendations for Aducanumab and not mentioned in the Lecanemab appropriate use recommendations. We will discuss how the current data may translate to its application in LBD and what needs to be considered when designing and interpreting trials for anti‐amyloid therapies in LBD.

Second, we will discuss how the presence of AD in LBD can also affect the effect of an intervention, even if it is not directed to AD pathology. In a recent Phase II study of neflamapimod, individuals with DLB and AD biomarkers were also less responsive to the experimental therapy. This warrants consideration of AD biomarkers in clinical trial design which will be discussed during the session.

Finally, we will comment on how the presence of copathology, specifically LBD, can also affect current AD‐specific clinical trials.

